# Heart failure in long-term survivors of childhood cancer – a systematic review and meta-analysis of population-based studies

**DOI:** 10.2340/1651-226X.2025.43654

**Published:** 2025-07-23

**Authors:** Tove Berg, Jens Böhmer, Bright I. Nwaru, Kristjan Karason, Marianne Jarfelt

**Affiliations:** a Department of Pediatric Medicine, The Queen Silvia Children’s Hospital, Sahlgrenska University Hospital, Gothenburg, Sweden; b Department of Pediatrics, Institute of Clinical Sciences, Sahlgrenska Academy, University of Gothenburg, Gothenburg, Sweden; c Department of Cardiology, Sahlgrenska University Hospital, Gothenburg, Sweden; d Department of Pediatrics, Frankfurt Hoechst Clinic, Frankfurt am Main, Germany; e Krefting Research Centre, Institute of Medicine, University of Gothenburg, Gothenburg, Sweden; f Transplant Institute, Sahlgrenska University Hospital, Gothenburg, Sweden; g Institute of Medicine, Sahlgrenska Academy, University of Gothenburg, Gothenburg, Sweden; h The Long-Term Follow Up Clinic for Childhood Cancer Survivors, Department of Oncology, Sahlgrenska University Hospital, Gothenburg, Sweden; i Department of Oncology, Institute of Clinical Sciences, Sahlgrenska Academy, University of Gothenburg, Gothenburg, Sweden

**Keywords:** Childhood cancer, survivors, heart failure, systematic review, meta-analysis

## Abstract

**Background and purpose:**

Heart failure is a well-recognised and serious non-malignant late complication among childhood cancer survivors. The primary aim of conducting this systematic review was to identify, critically appraise and synthesise population-based studies reporting on the incidence and/or prevalence of heart failure in 5-year survivors of childhood cancer (age < 18 years).

**Methods:**

We conducted this systematic review in accordance with the PRISMA (Preferred Reporting Items for Systematic Reviews and Meta-Analysis) guidelines. The protocol was registered in the International Prospective Register of Systematic Reviews (PROSPERO) in April 2021 (registration number: CRD42021247622) and published in June 2022. We searched databases Medline, Embase, Scopus, CINAHL, CAB International, AMED, Global Health, Psycinfo, Web of science and Google Scholar from their inception date until March 14, 2023. Screening, data extraction and quality assessment were conducted independently by two reviewers. The Effective Public Healthcare Practice Project tool was used for quality assessment.

**Results:**

Following a comprehensive review of the 3,883 records, only four were found to be eligible for inclusion. The overall quality of the studies was evaluated as strong in two studies and moderate in the remaining two studies. A subsequent meta-analysis of three comparable studies yielded a cumulative incidence of 0.99% (95% confidence interval [CI] 0.57–1.42) over an extended period of 5.0–72.5 years (*I*-squared = 94.4%, *p* < 0.001).

**Interpretation:**

Existing population-based studies reporting on heart failure in 5-year childhood cancer survivors are few and heterogeneous. Future population-based studies comparing heart failure incidence in childhood cancer survivors with the general population would be of significant value.

## Introduction

The survival rate after childhood cancer has improved significantly, with current figures exceeding 80% 5 years from diagnosis [1, 2], leading to a growing population of childhood cancer survivors (CCS). This development can be attributed to advancements in cancer treatment, risk stratification and supportive care [1]. However, it is concerning that approximately 40% of CCS continue to experience severe complications during long-term follow-up [3, 4]. The most prevalent non-malignant complications include cardiovascular diseases, particularly chemotherapy-induced cardiotoxicity, which can manifest as a range of conditions from asymptomatic left ventricular (LV) dysfunction to severe heart failure (HF) [5–8]. HF is a clinical syndrome where cardiac abnormalities give rise to noticeable symptoms and signs [9, 10, 11]. The clinical consequences of HF are serious, including impaired quality of life, frequent hospitalisations and increased risk of mortality [12].

Anthracyclines have been instrumental in elevating survival rates in paediatric cancer patients. However, it has been established that higher cumulative doses of these drugs are associated with an increased risk of cardiotoxicity [6, 13–17], with no established safe dose yet identified [15]. Notably, the addition of other types of chemotherapy agents to anthracyclines has the potential to enhance its cardiotoxicity [18]. Furthermore, the combination of anthracyclines with chest radiation has been identified as a significant risk factor for myocardial injury [13, 16, 19, 20]. In addition to chemotherapy-induced cardiotoxicity, the primary cancer itself and its associated treatment can both influence the biological mechanisms of ageing and increase the risk of developing cardiovascular risk factors [21]. Identification and management of modifiable risk factors such as hypertension, diabetes mellitus and dyslipidemia are crucial to delay cardiac dysfunction [22, 23]. In 2023, the International Late Effects of Childhood Cancer Guideline Harmonization Group (IGHG) published updated recommendations for cardiomyopathy surveillance in survivors [16]. The European Society of Cardiology (ESC) Guidelines on cardio-oncology (2022) also recommend screening for cardiovascular risk factors and suggest that regular echocardiographic surveillance should be considered after moderate and high-risk cardiotoxic treatments [24]. The incidence and prevalence of HF in the general population vary both globally and within Europe [12], in part due to differences in cardiovascular risk factors [25].

The term population based refers to studies that encompass the totality of cases of a particular disease within a specific region, which is of essential value when investigating the incidence and prevalence of a condition [26]. The strength of this design is that it reduces the risk of selection bias arising from subjective recruitment of participants [27]. In addition, such a design enables the generalisation of findings and can reveal the distribution and determinants of a disease in the population at large [28]. Consequently, the present systematic review has been narrowed to encompass exclusively population-based studies.

The primary aim of this systematic review was to identify, critically appraise and synthesise existing population-based studies reporting on the incidence of HF in 5-year survivors of childhood cancer. The secondary objectives of the study included identifying risk factors for delayed cardiotoxicity and exploring the potential relationship between cancer treatment across different eras and the development of late cardiac insufficiency.

## Methods

We developed a protocol in accordance with the recommendation of the Preferred Reporting Items for Systematic Reviews and Meta-Analysis (PRISMA-P) [29] and used the model of Participants, Interventions, Comparators, and Outcomes (PICO) to formulate the research questions [30].

### Eligibility criteria

We included population-based studies that reported the incidence of HF in patients, treated for any type of cancer before the age of 18 years, who had survived for a minimum of 5 years after their diagnosis. A study was considered population based if the patients included were representative of all cases of CCS occurring within a specified geographical area. Studies that did not meet the criteria for a population-based design, including randomised controlled trials, case series and case reports, were excluded. A diagnosis of HF was considered valid if given by a health care provider and recorded in a medical file, registry or database. Cases of self-reported HF were regarded credible if diagnosed by a physician or confirmed by medical records.

### Information sources and literature search strategy

We conducted this systematic review and meta-analysis in accordance with the PRISMA guidelines and the MOOSE standards (Meta-Analysis of Observational Studies in Epidemiology) [31, 32]. A literature search was performed in Medline, Embase, Scopus, CINAHL, CAB International, AMED, Global Health, PsycINFO, Web of Science and Google Scholar from their inception date until May 17, 2021, with an updated search on March 14, 2023. All keywords, including MeSH terms, related to *neoplasm*, *anthracyclines (and other types of treatment)*, *child*, *heart failure* and *epidemiology/survivors* were collected and used for the literature search, without restrictions based on language or publication status. The reference lists of retrieved papers were also screened. In articles lacking essential information, we reached out to the authors and asked if missing data could be retrieved. Our full search strategy is provided in Supplementary Material.

### Study selection and data extraction

Assessment of titles and abstracts of all retrieved articles, followed by full-text screening for potentially eligible papers, were performed independently by two reviewers (TB and JB). Any disagreement during the screening process was resolved by a discussion involving a third reviewer (MJ or KK). Methodological issues were resolved by BN, a statistician highly experienced in conducting systematic reviews. A standardised data extraction form was developed and tested as a pilot to make necessary adjustments before full data extraction was performed.

### Quality assessment

Two reviewers (TB and JB) used the Effective Public Healthcare Practice Project (EPHPP) [33] tool to score the quality and risk of bias in studies identified for the review. To receive an EPHPP score, the study is rated across six different domains: selection bias, study design, confounders, blinding, data collection methods and withdrawals and dropouts. Given that a number of studies in our systematic review lacked a control group and blinding was not feasible, we opted not to evaluate confounders and blinding, resulting in the quality being assessed across four domains. Taking this into account, the quality was classified as strong if there were no weak ratings in the four domains, moderate if there was one weak rating and weak if there were two or more weak ratings.

### Analysis/data synthesis

The meta-analysis was conducted using Stata statistical software version 15. For studies sufficiently homogenous with respect to their designs, populations, methods, interventions/exposures, outcomes and assessments, we implemented the random-effects meta-analysis using the method of DerSimonian and Laird in calculating the weights. We combined results from different studies by merging data on the cumulative incidence of HF. The cumulative incidence was calculated for the entire cohort and reported at the median follow-up time for studies that provided the total number of cases of HF at the end of the follow-up period. In the absence of data on the total number of cases of HF, the cumulative incidence at the longest follow-up time was employed as a substitute. When studies with overlapping data were identified, only one was included in the meta-analysis. The *I*-squared statistic was used to evaluate potential heterogeneity between studies. The small number and heterogeneity of papers included prevented us from exploring the influence of risk factors and treatment eras on incident HF as initially planned [30].

## Results

### Literature search and screening process

**Figure 1:**
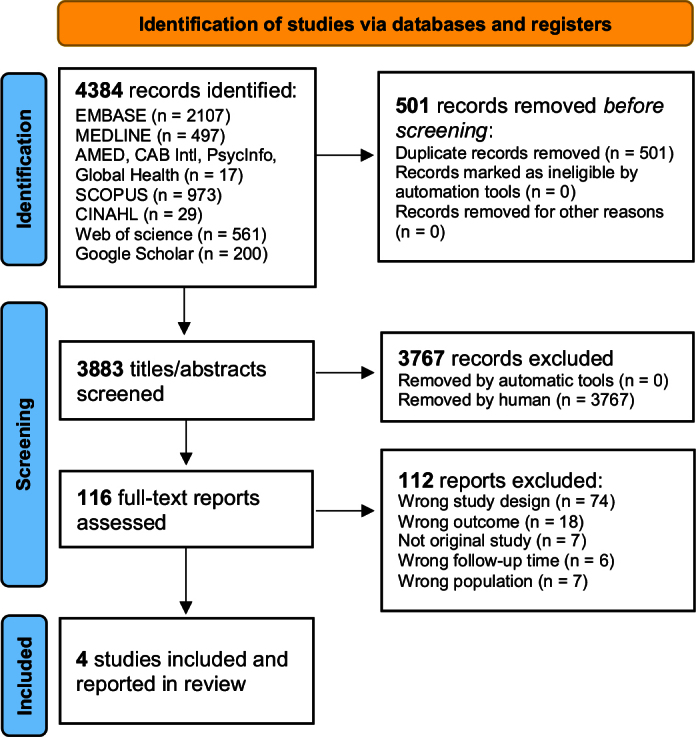
PRISMA flow diagram for selection of population-based studies reporting the incidence and/or prevalence of HF in 5-year CCS.

As outlined in our PRISMA flow diagram (Figure 1), the literature search identified 4,384 records, of which 3,883 papers remained after removal of duplicates. Following title and/or abstract screening, 3,767 records were excluded. After full-text screening of the remaining 116 papers, 112 reports were found to be ineligible based on the inclusion criteria. The remaining four papers, consisted of three published articles [34–36] and one research letter [37], all of which are included in the review.

Adapted to: Page MJ, McKenzie JE et al. The PRISMA 2020 statement: an updated guideline for reporting systematic reviews. BMJ. 2021.

### Study characteristics

Table 1 outlines the characteristics of the four studies included in the systematic review, covering study design, study population, inclusion criteria, age at cancer diagnosis, gender, cancer types, treatment era, definition of HF and age at follow-up. One study was conducted in the Netherlands [34], one in Canada [37] and one in Switzerland [36]. The remaining study was the PanCare Childhood and Adolescent Cancer Survivor Care and Follow-Up Studies (PanCareSurFup) cohort, which included eight sub-cohorts from seven European countries including France, Hungary, Italy (two sub-cohorts), the Netherlands, Slovenia, Switzerland and the United Kingdom [35]. However, the data from the French sub-cohort and the hospital-based sub-cohort from Italy were not considered population based and thus excluded from our systematic review. In total, the studies assessed in our analysis reported 45,111 CCSs diagnosed between 1940 and 2014. Nevertheless, there was a notable degree of overlap between the Dutch and Swiss cohorts studied by Feijen, Hau and the PanCareSurFup cohort studied by De Baat et al. [34–36]. All four studies included in this systematic review were cohort studies [34–37]. While two studies included a control group [36, 37], only one compared the outcome with matched individuals from the general population [37]. Three studies recruited survivors of all cancer types [34, 35, 37], while the remaining enrolled survivors of acute lymphoblastic leukaemia (ALL) [36]. Within the Swiss sub-cohort reported by De Baat et al., it is possible that a few individuals were older than the specified age limit of 18 years, given that the study included cancer patients up to 20 years old [35].

**Table 1:** Characteristics and outcomes of HF in 5-year CCS presented in population-based studies.

Reference, country; study design	Source(s) of study population and size		Inclusion criteria	Study population age at cancer diagnosis (years)	Male gender in study population *N* (%)	Cancer diagnoses	Treatment era	Data collection of HF outcome	Study population age at follow-up (years)	Cumulative incidence of HF (95% CI) or prevalence *N* (%)^a^ of HF at years from diagnosis^b^	Risk factors of HF	Overall quality
	Cases (*n*)	Controls (*n*)										
Feijen et al. 2019, The Netherlands; Nationwide cohort study	DCOG–LATER cohort (5,845)	No control group	5-year cancer survivors < 18 years, 1970–2001, included in the DCOG–LATER cohort	Median 5.5 (IQR, 2.8–10.5)	3,257 (55.7)	All cancer diagnoses	1970 – 2001	Collected from questionnaire, primary physician questionnaire and medical records. Graded according to the CTCAE	Median 27.3 (range 5.1–65.2)	Study population: 0.4% (0.2–0.5) at 10 years, 1.3% (1.0–1.7) at 20 years 3.0% (2.3–3.6) at 30 years, 4.4% (3.4–5.5) at 40 years, 1.98% (1.66–2.37) at a median of 19.9 years (range 5.0–50.4)	Treatment with mitoxantrone, cyclopho sphamide, anthracyclines, or RT involving the heart increased the risk for HF	Strong
Khanna et al. 2019, Canada; Population-based matched cohort study	The provincial paediatric cancer registry, POGONIS (7,289)	Cancer-free matched individuals from the general population (36,205)	5-year cancer survivors < 18 years, 1987–2010, treated in a paediatric centre in Ontario	Median 7.0 (range, 0 –17.9)	Not reported	All cancer diagnoses	1987–2010	Identified using established algorithms based on combinations of hospital admission and physician billing codes	Median 24 (range 5–47)	Study population: 1.1% (0.8–1.4) at 15 years 1.8% (1.4–2.3) at 20 years Matched controls: 0.1% (0.1–0.2) at 15 years 0.2% (0.1–0.2) at 20 years HR 9.7 (6.8–14.0)	Childhood relapse/subsequent cancer, exposure to ≥250 mg/m^2^ of doxorubicin equivalent anthracycline chemotherapy, diabetes and hypertension were statistically significant predictors of HF	Moderate
De Baat et al., 2022, Hungary, Italy, The Netherlands, Slovenia, Switzerland, United Kingdom; Seven Nationwide/population-based cohorts	Hungary: Hospital data, clinical trials, nationwide cancer registry (3,680), Italy: CCRP (1,514), The Netherlands: DCCSS LATER (5,185), Slovenia: Nationwide Slovenian cancer Registry follow-up clinic (1,147), Switzerland: Nationwide SCCR (3,176), United Kingdom: Nationwide cancer Registration (16,764), Total: (31,466)	No control group for HF outcome	5-year cancer survivors diagnosed: Hungary: <18 years, 1971–2001 Italy: <18 years, 1967–2005 The Netherlands: <18 years, 1964–2001 Slovenia: <16 years, 1961–2002 Switzerland: <20 years, 1964–2002 United Kingdom: <15 years, 1940–1991	Median (IQR) Hungary: 6.0 (3–11) Italy: 11.0 (4–17) The Netherlands: 6.0 (3–11) Slovenia: 8.0 (3–13) Switzerland: 6.0 (3–12) United Kingdom: 6.0 (3–10)	Hungary: 2060 (56) Italy: 779 (51) The Netherlands: 2885 (56) Slovenia: 642 (56) Switzerland: 1766 (56) United Kingdom: 9139 (55)	All Cancer diagnoses, Hungary including benign CNS tumours. Switzerland including LCH	Hungary: 1971–2001 Italy: 1967–2005 The Netherlands: 1964–2001 Slovenia: 1961–2002 Switzerland: 1964–2002 United Kingdom: 1940–1991	Identified using multiple strategies, for example, linkage to population-based databases and patient-based questionnaires. Symptomatic HF graded according to the CTCAE	Median (IQR) Hungary: 24 (18–30) Italy: 25 (19–32) The Netherlands: 28 (21–35) Slovenia: 30 (22–38) Switzerland: 22 (17–28) United Kingdom: 35 (27–44)	Study population: Hungary: 0.54% (0.35–0.84), range 5–35 years Italy: 1.45% (0.96–2.19), range 5–43 years The Netherlands: **97 (1.88)**, range 5–48 years Slovenia: 0.35% (0.14–0.89), range 5–52 years Switzerland: 0.25% (0.13–0.5), range 5–43 years UK: 0.79% (0.67–0.94), range 5–72 years Total cohort: **284 (0.9)** Median follow-up time 23.0 years for total PanCareSurFup-cohort	Case‑control study including 500 cases and 500 controls: A mean heart RT dose of 5 to <15 Gy increased the risk for HF. The risk of HF increased with the total cumulative anthracycline dose, there was no significantly increased risk of HF after treatment with a total anthracycline dose <100 mg/m^2^	Strong
Hau et al. 2019, Switzerland; Population-based cohort study	SCCR cohort (511)	Siblings of participating patients (1,299)	5-year survivors of ALL, diagnosed before age 16 years, 1976 – 2005 who were >16 years and alive at the time of the survey	0–4 (*N* = 244) 5–9 (*N* = 155) >10 (*N* = 112)	258 (50)	ALL	1976–2005	Self-reported, questionnaire, participants were asked whether a physician had ever told them that they had HF	16–20 (*N* = 135) 21–30 (*N* = 251) 31–40 (*N* = 107) >41 (*N* = 18)	Study population: **12 (2.35)** Siblings: **1 (0.08)** OR 13.9 (1.8–107.4) range 5–36 years, median follow-up time not reported	Not reported for HF specifically	Moderate

DCOG–LATER: Dutch Childhood Oncology Group – Long-Term Effects After Childhood Cancer; DCCSS LATER: Dutch Childhood Oncology Group Long-term effects registry; CCRP: Childhood Cancer Registry of Piedmont; SCCR: Swiss Childhood Cancer Registry; POGONIS: Pediatric Oncology Group of Ontario Networked Information System; CNS: Central Nervous System; LCH: Langerhans Cell Histiocytosis; HF: heart failure; CI: confidence interval; IQR: interquartile range.

a The prevalence N (%) is reported in bold text in instances where the cumulative incidence was not presented or calculated.

b The differences between study population and controls are presented in HR or OR (95% CI).

### Quality assessment

Table 1 displays the overall quality assessed by the EPHPP tool, with Table 2 showing the ratings of the specific components. In terms of the overall quality, two studies were rated as strong and two as moderate. The quality assessment of the study by De Baat et al. considered the ratings of all six sub-cohorts from the participating European countries [35].

**Table 2:** Domain-specific quality assessment and overall quality of population-based reporting on the incidence of HF in 5-year survivors of childhood cancer.

Reference	Selection bias	Study design	Data collection methods	Withdrawals and dropouts	Blinding*	Confounders*	Overall quality
Feijen et al. 2019	Strong	Moderate	Strong	Strong	Moderate	Not applicable	Strong
Khanna et al. 2019	Moderate	Moderate	Strong	Weak	Moderate	Weak	Moderate
De Baat et al. 2022	Strong	Moderate	Moderate	Moderate	Moderate	Not applicable	Strong
Hau et al. 2019	Moderate	Moderate	Weak	Moderate	Moderate	Strong	Moderate

Overall quality rating according to the EPHPP tool; STRONG (no WEAK ratings in individual domains), MODERATE (one WEAK rating), WEAK (two or more WEAK ratings).

* Not included in overall quality assessment.

### Data collection and outcome of HF in included studies

Data collection and validation of HF varied between studies (Table 1). Table 1 furthermore presents the reported cumulative incidence or prevalence of HF presented at years from follow-up (median follow-up time and range of follow-up time) when available in each study. Two studies collected information on the diagnosis of HF by questionnaire [34, 36]. In one of these studies, the diagnosis was validated against medical records and overt HF was considered to be present if it was graded 3–5 according to the Common Terminology Criteria for Adverse Events (CTCAE) [9, 34]. One of the studies identified HF cases using algorithms derived from hospital admission and physician billing codes [37]. Another identified potential cases by a linking information from population-based databases and administrating patient-based questionnaires, with HF diagnosed if the clinical state aligned with grade 3–5 based on CTCAE [35].

**Figure 2:**
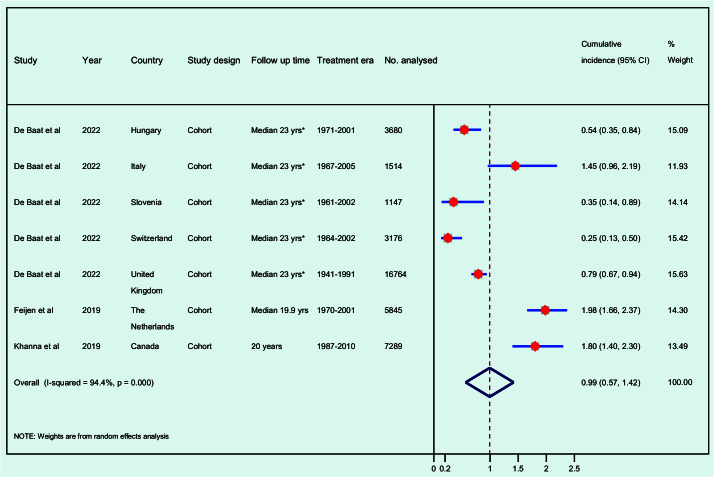
Meta-analysis. Cumulative incidence of heart failure in long-term childhood cancer survivors 5.0–72.5 years of follow-up after childhood cancer diagnosis. * The median follow-up time for the entire PanCareSurFup cohort is reported, as the median follow-up time for each individual cohort was unavailable. The range of follow-up periods for each sub-cohort is presented in Table 1.

Feijen and co-workers identified 116 cases of HF among 5,845 CCS during a median follow-up of 19.9 years, resulting in a cumulative incidence of 1.98% [34]. Khanna et al. studied 7,289 CCS and found that the cumulative incidence of HF was 1.1% after 15 years and 1.8% after 20 years from the survivor’s last paediatric cancer diagnosis. The corresponding rates for the cancer-free general population were 0.1% and 0.2%, resulting in an adjusted hazard ratio of 9.7 [37]. The PanCareSurFup study by De Baat and colleagues conducted in seven European countries calculated the overall cumulative incidence of HF (CTCAE grade 3–5) with attained age as the time scale. As shown in Table 1 and Figure 2, we used the reported data on new cases and cohort sizes from each country to estimate the cumulative incidence of HF for the six sub-cohorts separately. The ranges of follow-up time were calculated from the reported inclusion, based on years of diagnosis and the end-date of the study. The total study population was followed for a median of 23 years, but specific follow-up times for individual sub-cohorts were not provided [35]. Among 5-year survivors of ALL, Hau et al. identified 12 cases of HF (2.35%) and only one case among their siblings (0.08%), resulting in a weighted odds ratio of 13.9 (1.8–107.4) [36]. The median follow-up time was not reported.

### Meta-analysis of the cumulative incidence of HF

Three studies [34, 35, 37] on incident HF in long-term CCS were considered sufficiently homogenous to be included in the meta-analysis. The sub-populations in the study by De Baat et al. were treated as individual cohorts (Figure 2) with the exception of the Dutch sub-group, which was omitted due to an overlap with Feijen et al.’s cohort. We calculated the cumulative incidence at the end of the follow-up period for the studies by Feijen et al. and De Baat et al. Khanna et al. did not report the total number of HF cases, preventing the calculation of the cumulative incidence at the end of the follow-up period. Therefore, we relied on the cumulative incidence reported at 20 years from diagnosis, the longest follow-up time provided. The overall cumulative incidence of HF in the meta-analysis was 0.99% (95% confidence interval [CI] 0.57–1.42%) over a follow-up period ranging from 5.0 to 72.5 years after childhood cancer diagnosis. The *I*-squared statistic was 94.4% (*p* < 0.001) (Figure 2).

### Risk factors and treatment eras

The heterogeneity and low number of studies prevented us from analysing the proposed secondary outcomes in our meta-analysis. De Baat et al. performed a case‑control study for assessment of risk factors with 500 randomly selected controls who were matched 1:1 with 500 cases of HF [35]. Hau et al. provided no information on risk factors for HF in particular [36]. All three remaining studies observed a greater likelihood of developing HF after anthracycline treatment, with some reporting an increased risk with higher cumulative dosage [34, 35, 37]. Feijen et al. and De Baat et al. found that exposure of the heart during chest irradiation was a risk factor for HF [34, 35]. Other treatment-related or individual-level risk factors for HF identified are presented for each study in Table 1. Both Feijen et al. and De Baat et al. reported a higher cumulative incidence among CCS treated in more recent eras (1980–2001 and 1980–2008) as compared to earlier periods (1960–1979 and 1970–1979) [34, 35].

## Discussion

In this systematic review, we identified four population-based studies reporting on the risk for HF in long-term survivors of childhood cancer. The results from three similar studies were combined in a meta-analysis, revealing an overall cumulative incidence of 0.99% during a follow-up period ranging from 5.0 to 72.5 years. Notably, the *I*-squared statistic was high, indicating a considerable heterogenicity among the three studies. Still, this may also reflect a variation in the incidence of HF in CCS across different countries, similar to the general population [12].

Since we were unable to conduct a meta-analysis to address our secondary objectives, we derived conclusions from individual studies. All studies confirmed that exposure to higher doses of anthracyclines increased the risk for HF development [6, 16, 34, 35, 37]. Even low doses have been linked to cardiac abnormalities suggesting that no dosage can be considered safe for susceptible individuals [38–41]. Nevertheless, De Baat et al. found no increase in the risk of HF among survivors if the total anthracycline dose was less than 100 mg/m^2^ [35]. It is worth noting that cyclophosphamide and mitoxantrone are additional therapeutic agents that have been linked to increased HF risk independent of anthracycline exposure [34].

Radiotherapy (RT) with exposure of the heart is another established risk factor, especially in combination with anthracyclines [16, 20], as demonstrated in several of the included studies [34, 36, 37]. De Baat et al. reported that patients who received a mean heart RT dose of 5 to 15 Gy had an increased risk for HF. However, most participants were survivors of leukaemia, and radiation to the heart is likely to be part of total body irradiation, which can also increase the risk for metabolic syndrome [35, 42, 43].

Khanna et al. reported that diabetes and hypertension were predictors of HF in CCS [37]. Also, a study based on CCSS data showed that the presence of cardiovascular risk factors, especially hypertension, significantly increased the risk for HF [22]. These risk factors correspond to those related to HF in the general population [10, 12], but CCSs are likely to be more susceptible [21, 22]. In addition, CCSs have been reported to have higher rates of hypertension than matched controls [44]. Apart from cancer treatment and lifestyle factors, genetic variants have been associated with an increased risk of anthracycline-induced cardiotoxicity [45, 46]. A family history of cardiovascular disease independently increased the risk of both hypertension and HF in CCS exposed to chest-directed radiation and/or anthracycline chemotherapy in a report from SJLIFE [47].

Findings from the studies included in this systematic review showed an increased risk for HF among patients treated in the more recent treatment periods compared to earlier periods [34, 35]. This may be attributed to changes in the treatment regimen over time. To gain better understanding of this trend, it would be interesting to compare changes in HF incidence over time with the that in the general population. This is of particular interest given the increasing incidence of HF in young adults within the general population. This phenomenon could be partly explained by changes in coding practices, but it may also suggest a change in phenotype, with an increasing proportion of HF due to cardiomyopathy [48, 49].

Overall, the risk factors identified in this systematic review were consistent with those reported in previous studies [17, 20, 22, 23]. They are also in line with the updated IGHG and ESC guidelines on cardio-oncology, which incorporate a more comprehensive assessment of HF risk factors [16, 24]. Based on the findings of our systematic review, we consider the current recommendations to be appropriate.

## Strengths and limitations

The strength of this study is the well-defined inclusion criteria, structured literature search, screening, and synthesis in accordance with the PRISMA-guidelines. The results presented in this systematic review and meta-analysis only answer the primary question of this study, which concerned the incidence of HF in CCS in population-based studies.

One limitation of our review is that we, somewhat unexpectedly, identified only four studies that met our inclusion criterion. The limited number of eligible studies, along with their methodological heterogeneity, posed challenges for synthesis and hindered our ability to draw robust conclusions regarding the risk of HF in this population. However, as our overarching goal was to include only population-based studies as the most appropriate to estimate incidence of the outcome, expanding the number of studies beyond those that met our predefined inclusion criteria would have deviated from our original aim. Synthesising the results of the studies included in this systematic review was challenging due to differences in study cohorts, presentation of HF outcomes and data collection, which affected the estimation of HF incidence [50]. Hau et al. included only ALL survivors, which makes comparison difficult due to differences in cancer treatment and associated HF risks. Consequently, the study performed by Hau et al. was excluded from the meta-analysis [13, 19, 23]. Although the median follow-up time and age at cancer diagnosis were similar across the studies, the range of these parameters varied. For instance, Feijen and Khanna et al. included survivors from 5 years of age [34, 37], while Hau and De Baat included survivors above the age of 16 [35, 36]. When interpreting the cumulative incidence of HF in CCS, it is imperative to consider the follow-up time and age at follow-up, since the cumulative incidence increases over time, as reported in the included studies [34, 35, 37].

Certain high-quality studies on incident HF in CCS were excluded due to a non-population-based design, such as the North American Childhood Cancer Survivor Study (CCSS) [7, 22, 51], the St. Jude Lifetime Cohort Study (SJLIFE) [52, 53] and the German Cardiac and Vascular late Sequelae in long-term Survivors of childhood cancer (CVSS) study [54]. However, research based on CCSS data have reported a variation of HF rates, due to variations in different sub-cohorts or endpoints, similar to the papers included in this systematic review [7, 17, 22, 51]. Three population-based studies reporting cases of HF requiring hospitalisation were excluded since failing to account for outpatients would probably lead to underestimation of the true incidence [55–57]. Furthermore, one of these studies did not differentiate between 5-year survivors and those who had a shorter lifespan from cancer diagnosis [56]. Another study from Canada on survivors of ALL and acute myeloblastic leukaemia (AML) was excluded for this same reason [58]. A recent survey covering the entire Swedish population found that individuals under 25 years who were diagnosed with cancer between 1958 and 2021 had a 1.66 increased likelihood of experiencing HF or cardiomyopathy as compared to a matched control group from the general population. However, information on 5-year survivors was not provided [59]. A systematic review published in January 2024 on late mortality among CCS indicated that the risk of late mortality may differ significantly between world regions. However, the reasons for these regional differences remain unclear [60].

## Conclusions

The major findings of this systematic review are the low number of population-based studies of HF in 5-year CCS and a non-homogenous structure of both cohort definitions and HF outcome measures. This makes it challenging to obtain a comprehensive understanding of the frequency of HF in long-term CCS in general but also in various sub-populations. Therefore, future population-based studies comparing HF incidence in CCS with the general population would be of significant value. The acquisition of such epidemiological knowledge allowing for the estimation of distributions and prevalence rates and to assess risk factors over time is likely to be of value when generating future treatment protocols and organising cardiac surveillance programmes for CCS.

## Supplementary Material



## Data Availability

All data generated and analysed during the course of this study are included in this publication (along with its supplementary material).
