# Supplementary material for: Heart failure in long-term survivors of childhood cancer – a systematic review and meta-analysis of population-based studies
Source: Acta Oncol. 2025 Jul 23;64:43654. doi: 10.2340/1651-226X.2025.43654 (PMC12322935; doi:10.2340/1651-226X.2025.43654)
Supplement: Supplementary file 1 [file AO-64-43654-s1.pdf]

Supplementary material has been published as submitted. It has not been copyedited, or typeset by Acta Oncologica

Heart failure in long-term survivors of childhood cancer – Systematic review

DATABASE SEARCHES

1TH JUNE 2021

Search Terms	Number of retrieved records						
	EMBASE	MEDLINE	AMED, CAB Intl, PsycInfo, Global Health	SCOPUS	CINAHL	Web of Science	Google Scholar
1. neoplasm.mp. or exp Neoplasms/ or tumor.mp. or tumors.mp. or tumour.mp. or tumours.mp. or leukemia.mp. or exp Leukemia/ or exp Leukemia, Myelomonocytic, Acute/ or exp Leukemia, Myelomonocytic, Juvenile/ or exp Leukemia, B-Cell/ or exp Leukemia, T-Cell/ or Leukemia/ or exp Leukemia, Lymphoid/ or exp Leukemia, Myeloid/ or exp Leukemia, Myeloid, Acute/ or exp Precursor T-Cell Lymphoblastic Leukemia-Lymphoma/ or exp Precursor Cell Lymphoblastic Leukemia-Lymphoma/ or exp Precursor B-Cell Lymphoblastic Leukemia-Lymphoma/ or neoplasia.mp. or neoplasias.mp. or cancer.mp. or cancers.mp. or malignancy.mp. or malignancies.mp. or Malignant Neoplasms.mp. or Malignant Neoplasms.mp.	5,889,007	4,181,089	749,797	9,330,483	199,853	4,005,925	
2. Anthracyclines.mp. or exp Anthracyclines/ or radiation.mp. or exp Radiation/ or radiotherapy.mp. or exp Radiotherapy/ or "radiation therapy".mp. or "antineoplastic agents".mp. or exp Antineoplastic Agents/ or "antineoplastic therapy".mp. or Anthracycline.mp. or exp Anthracyclines/ or aclarubicin.mp. or exp Aclarubicin/ or exp Doxorubicin/ or exp Daunorubicin/ or danorubicin.mp. or carubicin.mp. or exp Carubicin/ or idarubicin.mp. or exp Idarubicin/ or nogalamycin.mp. or exp Nogalamycin/ or plicamycin.mp. or exp Plicamycin/ or "Anticancer Agent".mp. or exp Antineoplastic Agents/ or "Antineoplastic Drugs".mp. or Antineoplastic.mp. or "Antitumor Drug".mp. or "Antitumor Drugs".mp. or "Cancer Chemotherapy Agent".mp. or Antineoplastics.mp. or "Antitumor Agents".mp. or "Cancer Chemotherapy Drugs".mp. or mitoxantrone.mp. or exp Mitoxantrone/ or Acetate.mp. or exp Acetates/ or Hydrochloride.mp. or Novantrone.mp. or	3,745,324	2,326,902	603,654	236,048	64,100	1,609,550	

exp Antineoplastic Combined Chemotherapy Protocols/ or exp Radiotherapy, Intensity-Modulated/ or exp Radiotherapy, Conformal/ or exp Radiotherapy, Adjuvant/ or exp Radiotherapy Dosage/							200
3. child.mp. or exp Child/ or children.mp. or pediatric.mp. or exp Pediatrics/ or exp Infant/ or exp Child, Preschool/ or paediatric.mp. or childhood.mp. or exp Adolescent/ or adolescent.mp. or adolescents.mp. or baby.mp. or exp Infant, Newborn/ or infancy.mp. or infanthood.mp. or toddler.mp. or underage.mp. or early life.mp.	3,965,048	3,931,327	1,917,930	5,465,476	260,774	2,696,226	
4. 1 and 2 and 3	165,053	104,261	4,363	30,131	2,734	26,279	
5. "heart failure".mp. or exp Heart Failure/ or "Heart failure diastolic".mp. or exp Heart Failure, Diastolic/ or "Heart failure systolic".mp. or exp Heart Failure, Systolic/ or "heart failure left sided".mp. or "left sided heart failure".mp. or "Heart failure right-sided".mp. or "right sided heart failure".mp. or Cardiomyopathy.mp. or Cardiomyopathies/ or "myocardial failure".mp. or "congestive heart failure".mp. or "heart decompensation".mp. or "cardiac failure".mp. or "left ventricular heart failure".mp. or exp Ventricular Dysfunction, Left/ or exp Ventricular Dysfunction, Right/ or "right ventricular heart failure".mp. or "decompensation heart".mp. or "myocardial disease".mp. or "myocardial disorder".mp. or "myocardial diseases".mp. or "myocardial disorders".mp. or myocardiopathy.mp. or myocardiopathies.mp. or exp Myocardium/ or exp Cardiomyopathies/ or exp Myocytes, Cardiac/ or exp Cardiomyopathy, Hypertrophic/	767,049	475,574	46,334	1,215,337	29,128	426,461	
6. mortality.mp. or exp Mortality/ or death.mp. or exp Death/ or "mortality rate".mp. or "death rate".mp. or "mortality rates".mp. or "death rates".mp. or "excess mortality".mp. or "fatality rate".mp. or incidence.mp. or exp Incidence/ or "incidence rate".mp. or "incidence rates".mp. or exp Prevalence/ or prevalence.mp. or "prevalence rate".mp. or "prevalence rates".mp. or "incidence proportion".mp. or "point prevalence".mp or "period prevalence".mp or "life-time prevalence".mp or "cumulative incidence".mp or Long-Term Cancer Survivors.mp. or exp Cancer Survivors/ or Survivors, Cancer.mp. or Cancer Survivor, Long-Term.mp. or Cancer Survivors, Long-Term.mp. or Long Term Cancer Survivors.mp. or Long-Term Cancer Survivor.mp. or Survivor, Long-Term Cancer.mp. or Survivors, Long-Term Cancer.mp. or Survivors of Childhood Cancer.mp. or Cancer Survivor, Childhood.mp. or Cancer Survivors, Childhood.mp. or Childhood Cancer Survivor.mp. or Childhood Cancer Survivors.mp.	4,532,814	2,918,515	2,022,359	9,970,852	198,226	3,302,367	
7. 5 and 6	294,977	111,985	14,349	635,057	8,925	121,678	
8. 4 and 7	1,726	465	15	832	29	492	
Updated 14th March 2023 (17th May 2021 to 14th March 2023)<br>**limit 8 to dt=20210517-20230314 (EMBASE)**<br>**limit 8 to dt=20210517-20230314 [May 17th, 2021 to March 14th, 2023] (MEDLINE)**	381	32	2	141	0	69	

**TOTAL BEFORE DUPLICATES ARE REMOVED**	**3,759**
**TOTAL BEFORE DUPLICATES ARE REMOVED (updated searches)**	**625**
**TOTAL TO SCREEN AFTER DUPLICATES ARE REMOVED**	**3,327**
**TOTAL TO SCREEN AFTER DUPLICATES ARE REMOVED (updated searches)**	**556**

SCOPUS FULL SEARCH STRATEGY

1. ALL(Neoplasm or Neoplasms or tumor or tumors or tumour or tumours or leukemia or Leukemia or “Acute Myelomonocytic Leukemia” or “Juvenile Myelomonocytic Leukemia” or “B-Cell Leukemia” or “T-Cell Leukemia” or “Lymphoid Leukemia” or “Myeloid Leukemia” or “Acute Myeloid Leukemia” or “Precursor T-Cell Lymphoblastic Leukemia-Lymphoma” or “Precursor Cell Lymphoblastic Leukemia-Lymphoma” or “Precursor B-Cell Lymphoblastic Leukemia-Lymphoma” or neoplasia or neoplasias or cancer or cancers or malignancy or malignancies or “Malignant Neoplasms”)
2. ALL(Anthracyclines or Anthracyclines or radiation or Radiation or radiotherapy or Radiotherapy or "radiation therapy" or "antineoplastic agents" or “Antineoplastic Agents” or "antineoplastic therapy" or Anthracycline or Anthracyclines or aclarubicin or Aclarubicin or Doxorubicin or Daunorubicin or danorubicin or carubicin or Carubicin or idarubicin or Idarubicin or nogalamycin or Nogalamycin or plicamycin or Plicamycin or "Anticancer Agent" or "Antineoplastic Drugs" or Antineoplastic or "Antitumor Drug" or "Antitumor Drugs" or "Cancer Chemotherapy Agent" or Antineoplastics or "Antitumor Agents" or "Cancer Chemotherapy Drugs" or mitoxantrone or Mitoxantrone or Acetate or Acetates or Hydrochloride or Novantrone or Antineoplastic “Combined Chemotherapy Protocols” or “Intensity-Modulated Radiotherapy” or “Conformal Radiotherapy” or “Adjuvant Radiotherapy” or “Radiotherapy Dosage”)
3. ALL(child or Child or children or pediatric or Pediatrics or Infant or Child, Preschool or paediatric or childhood or Adolescent or adolescent or adolescents or baby or Newborn or infancy or infanthood or toddler or underage or “early life”)
4. #1 AND #2 AND #3
5. ALL("heart failure" or “Heart Failure” or "Heart failure diastolic" or “Diastolic Heart Failure” or "systolic Heart failure" or "left sided heart failure" or "right sided heart failure" or Cardiomyopathy or Cardiomyopathies or "myocardial failure" or "congestive heart failure" or "heart decompensation" or "cardiac failure" or "left ventricular heart failure" or “Left Ventricular Dysfunction” or “Right Ventricular Dysfunction” or "right ventricular heart failure" or "decompensation heart" or "myocardial disease" or "myocardial disorder" or "myocardial diseases" or "myocardial disorders" or myocardiopathy or myocardiopathies or Myocardium or Cardiomyopathies or “Cardiac Myocytes” or “Hypertrophic Cardiomyopathy”)

6. ALL(mortality or Mortality or death or Death or "mortality rate" or "death rate" or "mortality rates" or "death rates" or "excess mortality" or "fatality rate" or incidence or Incidence or "incidence rate" or "incidence rates" or Prevalence or prevalence or "prevalence rate" or "prevalence rates" or "incidence proportion" or "point prevalence" or "period prevalence" or "life-time prevalence" or "cumulative incidence" or “Long-Term Cancer Survivors” or “Cancer Survivors” or “Cancer Survivor” or “Long-Term Cancer Survivor” or “Survivors of Childhood Cancer” or “Childhood Cancer Survivor” or “Childhood Cancer Survivors”)
7. #5 AND #6
8. #4 AND #7

CINAHL FULL SEARCH STRATEGY

S1. ALL(Neoplasm or Neoplasms or tumor or tumors or tumour or tumours or leukemia or Leukemia or “Acute Myelomonocytic Leukemia” or “Juvenile Myelomonocytic Leukemia” or “B-Cell Leukemia” or “T-Cell Leukemia” or “Lymphoid Leukemia” or “Myeloid Leukemia” or “Acute Myeloid Leukemia” or “Precursor T-Cell Lymphoblastic Leukemia-Lymphoma” or “Precursor Cell Lymphoblastic Leukemia-Lymphoma” or “Precursor B-Cell Lymphoblastic Leukemia-Lymphoma” or neoplasia or neoplasias or cancer or cancers or malignancy or malignancies or “Malignant Neoplasms”)

S2. ALL(Anthracyclines or Anthracyclines or radiation or Radiation or radiotherapy or Radiotherapy or "radiation therapy" or "antineoplastic agents" or “Antineoplastic Agents” or "antineoplastic therapy" or Anthracycline or Anthracyclines or aclarubicin or Aclarubicin or Doxorubicin or Daunorubicin or danorubicin or carubicin or Carubicin or idarubicin or Idarubicin or nogalamycin or Nogalamycin or plicamycin or Plicamycin or "Anticancer Agent" or "Antineoplastic Drugs" or Antineoplastic or "Antitumor Drug" or "Antitumor Drugs" or "Cancer Chemotherapy Agent" or Antineoplastics or "Antitumor Agents" or "Cancer Chemotherapy Drugs" or mitoxantrone or Mitoxantrone or Acetate or Acetates or Hydrochloride or Novantrone or Antineoplastic “Combined Chemotherapy Protocols” or “Intensity-Modulated Radiotherapy” or “Conformal Radiotherapy” or “Adjuvant Radiotherapy” or “Radiotherapy Dosage”)

S3. ALL(child or Child or children or pediatric or Pediatrics or Infant or Child, Preschool or paediatric or childhood or Adolescent or adolescent or adolescents or baby or Newborn or infancy or infanthood or toddler or underage or “early life”)

S4. S1 AND S2 AND S3

S5. ALL("heart failure" or “Heart Failure” or "Heart failure diastolic" or “Diastolic Heart Failure” or "systolic Heart failure" or "left sided heart failure" or "right sided heart failure" or Cardiomyopathy or Cardiomyopathies or "myocardial failure" or "congestive heart failure" or "heart decompensation" or "cardiac failure" or "left ventricular heart failure" or “Left Ventricular Dysfunction” or “Right Ventricular Dysfunction” or "right ventricular heart failure" or

"decompensation heart" or "myocardial disease" or "myocardial disorder" or "myocardial diseases" or "myocardial disorders" or myocardiopathy or myocardiopathies or Myocardium or Cardiomyopathies or “Cardiac Myocytes” or “Hypertrophic Cardiomyopathy”)

S6. ALL(mortality or Mortality or death or Death or "mortality rate" or "death rate" or "mortality rates" or "death rates" or "excess mortality" or "fatality rate" or incidence or Incidence or "incidence rate" or "incidence rates" or Prevalence or prevalence or "prevalence rate" or "prevalence rates" or "incidence proportion" or "point prevalence" or "period prevalence" or "life-time prevalence" or "cumulative incidence" or “Long-Term Cancer Survivors” or “Cancer Survivors” or “Cancer Survivor” or “Long-Term Cancer Survivor” or “Survivors of Childhood Cancer” or “Childhood Cancer Survivor” or “Childhood Cancer Survivors”)

S7. S5 AND S6
S8. S4 AND S7

WEB OF SCIENCE FULL SEARCH STRATEGY

1. TS=(Neoplasm or Neoplasms or tumor or tumors or tumour or tumours or leukemia or Leukemia or “Acute Myelomonocytic Leukemia” or “Juvenile Myelomonocytic Leukemia” or “B-Cell Leukemia” or “T-Cell Leukemia” or “Lymphoid Leukemia” or “Myeloid Leukemia” or “Acute Myeloid Leukemia” or “Precursor T-Cell Lymphoblastic Leukemia-Lymphoma” or “Precursor Cell Lymphoblastic Leukemia-Lymphoma” or “Precursor B-Cell Lymphoblastic Leukemia-Lymphoma” or neoplasia or neoplasias or cancer or cancers or malignancy or malignancies or “Malignant Neoplasms”)
2. 2. TS=(Anthracyclines or Anthracyclines or radiation or Radiation or radiotherapy or Radiotherapy or "radiation therapy" or "antineoplastic agents" or “Antineoplastic Agents” or "antineoplastic therapy" or Anthracycline or Anthracyclines or aclarubicin or Aclarubicin or Doxorubicin or Daunorubicin or danorubicin or carubicin or Carubicin or idarubicin or Idarubicin or nogalamycin or Nogalamycin or plicamycin or Plicamycin or "Anticancer Agent" or "Antineoplastic Drugs" or Antineoplastic or "Antitumor Drug" or "Antitumor Drugs" or "Cancer Chemotherapy Agent" or Antineoplastics or "Antitumor Agents" or "Cancer Chemotherapy Drugs" or mitoxantrone or Mitoxantrone or Acetate or Acetates or Hydrochloride or Novantrone or Antineoplastic “Combined Chemotherapy Protocols” or “Intensity-Modulated Radiotherapy” or “Conformal Radiotherapy” or “Adjuvant Radiotherapy” or “Radiotherapy Dosage”)
3. TS=(child or Child or children or pediatric or Pediatrics or Infant or Child, Preschool or paediatric or childhood or Adolescent or adolescent or adolescents or baby or Newborn or infancy or infanthood or toddler or underage or “early life”)
4. #1 AND #2 AND #3
5. TS=("heart failure" or “Heart Failure” or "Heart failure diastolic" or “Diastolic Heart Failure” or "systolic Heart failure" or "left sided heart failure" or "right sided heart failure" or Cardiomyopathy or Cardiomyopathies or "myocardial failure" or "congestive heart failure" or "heart decompensation" or "cardiac failure" or "left ventricular heart failure" or “Left Ventricular Dysfunction” or “Right Ventricular Dysfunction” or "right ventricular heart failure" or "decompensation heart" or "myocardial disease" or "myocardial disorder" or "myocardial diseases" or "myocardial disorders" or myocardiopathy or myocardiopathies or Myocardium or Cardiomyopathies or “Cardiac Myocytes” or “Hypertrophic Cardiomyopathy”)

6. TS=(mortality or Mortality or death or Death or "mortality rate" or "death rate" or "mortality rates" or "death rates" or "excess mortality" or "fatality rate" or incidence or Incidence or "incidence rate" or "incidence rates" or Prevalence or prevalence or "prevalence rate" or "prevalence rates" or "incidence proportion" or "point prevalence" or "period prevalence" or "life-time prevalence" or "cumulative incidence" or “Long-Term Cancer Survivors” or “Cancer Survivors” or “Cancer Survivor” or “Long-Term Cancer Survivor” or “Survivors of Childhood Cancer” or “Childhood Cancer Survivor” or “Childhood Cancer Survivors”)
7. #5 AND #6
8. #4 AND #7

GOOGLE SCHOLAR
((neoplasm OR cancer OR tumor OR leukemia OR malignancy) AND (treatment OR chemotherapy OR radiation OR radiotherapy) AND (child OR pediatric OR baby OR infant OR adolescent)) AND ((“heart failure” OR cardiomyopathy OR “myocardial failure” OR myocytes OR myocardium) AND (death OR mortality OR incidence OR survivor OR prevalence))
